# Preservation of micro-architecture and angiogenic potential in a pulmonary acellular matrix obtained using intermittent intra-tracheal flow of detergent enzymatic treatment

**DOI:** 10.1016/j.biomaterials.2013.05.015

**Published:** 2013-09

**Authors:** Panagiotis Maghsoudlou, Fanourios Georgiades, Athanasios Tyraskis, Giorgia Totonelli, Stavros P. Loukogeorgakis, Giuseppe Orlando, Panicos Shangaris, Peggy Lange, Jean-Marie Delalande, Alan J. Burns, Angelo Cenedese, Neil J. Sebire, Mark Turmaine, Brogan N. Guest, John F. Alcorn, Anthony Atala, Martin A. Birchall, Martin J. Elliott, Simon Eaton, Agostino Pierro, Thomas W. Gilbert, Paolo De Coppi

**Affiliations:** a Surgery Unit, UCL Institute of Child Health and Great Ormond Street Hospital, 30 Guilford Street, London WC1N 1EH, UK; b Wake Forest Institute for Regenerative Medicine, Wake Forest University School of Medicine, Winston-Salem, NC 27157, USA; c Neural Development Unit, UCL Institute of Child Health, London WC1N 1EH, UK; d Department of Information Engineering, University of Padua, Italy; e Department of Histopathology, UCL Institute of Child Health and Great Ormond Street Hospital, London WC1N 1EH, UK; f Division of Bioscience, University College London, London WC1N 1EH, UK; g McGowan Institute for Regenerative Medicine, Department of Surgery, University of Pittsburgh, Pittsburgh, PA, USA; h Department of Pediatrics, Children's Hospital of Pittsburgh of UPMC, Pittsburgh, PA, USA; i UCL Ear Institute, London WC1X 8EE, UK; j Department of Cardiothoracic Surgery, Great Ormond Street Hospital, London WC1N 3JH, UK; k Department of Cardiothoracic Surgery, Children's Hospital of Pittsburgh of UPMC, Pittsburgh, PA, USA

**Keywords:** Decellularization, Natural acellular scaffold, Lung tissue engineering, Extracellular matrix, Angiogenesis

## Abstract

Tissue engineering of autologous lung tissue aims to become a therapeutic alternative to transplantation. Efforts published so far in creating scaffolds have used harsh decellularization techniques that damage the extracellular matrix (ECM), deplete its components and take up to 5 weeks to perform. The aim of this study was to create a lung natural acellular scaffold using a method that will reduce the time of production and better preserve scaffold architecture and ECM components. Decellularization of rat lungs via the intratracheal route removed most of the nuclear material when compared to the other entry points. An intermittent inflation approach that mimics lung respiration yielded an acellular scaffold in a shorter time with an improved preservation of pulmonary micro-architecture. Electron microscopy demonstrated the maintenance of an intact alveolar network, with no evidence of collapse or tearing. Pulsatile dye injection via the vasculature indicated an intact capillary network in the scaffold. Morphometry analysis demonstrated a significant increase in alveolar fractional volume, with alveolar size analysis confirming that alveolar dimensions were maintained. Biomechanical testing of the scaffolds indicated an increase in resistance and elastance when compared to fresh lungs. Staining and quantification for ECM components showed a presence of collagen, elastin, GAG and laminin. The intratracheal intermittent decellularization methodology could be translated to sheep lungs, demonstrating a preservation of ECM components, alveolar and vascular architecture. Decellularization treatment and methodology preserves lung architecture and ECM whilst reducing the production time to 3 h. Cell seeding and in vivo experiments are necessary to proceed towards clinical translation.

## 1 Introduction

Chronic obstructive pulmonary disease (COPD) is the fifth largest cause of death with a worldwide mortality of 3 million people annually. Estimates from the World Health Organization indicate that by 2030, COPD will become the third leading cause of death [1]. Due to the poor regenerative capability of the lung, the only definitive treatment is lung transplantation. However, acute rejection, graft failure and the need for immunosuppression lead to a survival of 79% at 1 year, 53% at 5 years, and 30% at 10 years [2]. The development of a tissue-engineered lung that could be transplanted without the need for immunosuppression would provide a therapeutic alternative.

Tissue engineering has already found clinical applications in a number of organs including the bladder [3], urethra [4], and trachea, both in adults [5,6] and children [7]. However engineering of complex organs is still limited. When comparing the trachea to the lung, the latter is more intricate both in function and in structure leading to greater challenges in its engineering. For this reason, a handful of efforts in animal models have been unable to yield considerable progress. The intricacy of the alveolar network, the alveolus-capillary interface along with the variety of cell types that populate it, render it difficult to replicate this architecture from the micro- to the macro-scale. From the material science aspect, three kinds of scaffold have been used; namely naturally-derived, synthetic, and natural acellular matrices.

Naturally-derived materials have included collagen [8], matrigel [9], and Gelfoam [10], the latter being a porous form of porcine gelatin. When Andrade [10] used Gelfoam as a scaffold for fetal rat lung cells, allowing 7 days of in vitro growth prior to injecting the construct into the lung parenchyma they demonstrated the appearance of alveolar-like structures that stained positive for pro-surfactant protein-C (SP-C), Clara cell secreted protein (CC-10) and Von Willebrand factor. The regenerated alveoli however, were present only in the periphery of the sponge and stained poorly for CellTracker orange, the dye that was used to follow the transplanted cells. When the pulmonary artery was injected with India Ink to evaluate vascularization of the construct there was no evidence of formation of the alveolus-capillary junctions needed for gas exchange. This study re-affirms the need for a scaffold with an inherent pulmonary-like micro-architecture.

Synthetic scaffolds that have been employed for lung tissue engineering include PGA-F127 [11], PLA, and PLGA [12]. Cortiella et al. [11] have combined ovine somatic lung progenitor cells (SLPC) and a PGA/PF-127 scaffold and implanted the construct on the backs of nude mice. The constructs developed into alveolar-like structures that were positive for SP-C, CC-10, collagen and smooth muscle actin. When these constructs were placed in the space left following a pneumonectomy in sheep, fleshy vascularized tissue developed, which however lacked any development of alveolar architecture or composition despite having adequate blood supply.

The use of natural acellular matrices could solve the issues that have been encountered so far with animal studies for lung regeneration, since in an optimally decellularized lung the hierarchical pulmonary network and alveolus-capillary junctions would be preserved. Ott et al. [13] perfused rat lungs via the pulmonary artery with 0.1% sodium dodecyl sulfate (SDS) (120 min), 1% Triton-X (10 min) and phosphate buffed saline with antibiotic and antimycotic (PBS/AA) (72 h), a total of 74 h, to obtain decellularized scaffolds. The scaffolds were seeded with rat fetal lung cells, and following a left pneumonectomy, were transplanted orthotopically in rats. Rats with transplanted constructs developed pulmonary edema following extubation when compared to pneumonectomized controls, even though arterial oxygen tension was increased. Petersen et al. [14] performed rat lung decellularization via both the pulmonary artery and trachea using a solution of 8 mm CHAPS, 1m NaCl, and 25 mm EDTA, followed by PBS/AA, 90 U/ml endonuclease and PBS containing 10% FBS, with the complete protocol lasting up to 5 weeks. The scaffold supported cell growth following seeding with neonatal rat lung epithelial cells and microvascular lung endothelial cells. When implanted into rats in vivo for short time intervals (up to 120 min) the engineered lungs participated in gas exchange even though they inflated less than the native right lung and bleeding occurred in the airways.

Both of the above approaches need refining since they use long decellulization protocols that would be impractical and difficult to replicate in humans. Moreover, decellularization protocols that include extensive use of detergent may lead to the loss of important information contained in the native tissue. For example, SDS is known to disrupt tissue ultrastructure [15] and eliminate growth factors [16]. The development of a brief decellularization technique that retains ECM architecture without removing growth factors would be paramount for further in vivo work.

## 2 Materials and methods

### 2.1 Harvest of organs

A midline thoracotomy was performed, the muscles of the head and neck dissected away from the midline and the trachea sectioned above the cricoid cartilage. The thymus was removed and the pulmonary artery (PA) cannulated via the right atrium, secured with sutures, flushed with PBS/AA and the inferior and superior vena cavae were transected. The lung was mobilized from the cricoid cartilage and dissected free from its attachments to the esophagus and thoracic cavity.

### 2.2 Decellularization

The lung was decellularized using four protocols:

#### 2.2.1 Protocol A (IV)

The pulmonary artery was perfused with continuous fluid delivery using a Masterflex L/S variable speed roller pump at 0.6 ml/min. Each DET cycle was composed of de-ionized water (resistivity 18.2 mΩ/cm) at 4 °C for 24 h, 4% sodium deoxycholate (Sigma) at room temperature (RT) for 4 h, and 2000kU DNase-I (Sigma) in 1m NaCl (Sigma) at RT for 3 h. Up to 4 cycles were carried out.

#### 2.2.2 Protocol B (IT/IV)

The trachea and pulmonary artery were perfused with continuous fluid delivery using a Masterflex L/S variable speed roller pump at 0.6 ml/min. Each DET cycle was composed of de-ionized water (resistivity 18.2 mΩ/cm) at 4 °C for 24 h, 4% sodium deoxycholate (Sigma) at room temperature (RT) for 4 h, and 2000kU DNase-I (Sigma) in 1m NaCl (Sigma) at RT for 3 h. Up to 4 cycles were carried out.

#### 2.2.3 Protocol C (IT)

The trachea was perfused with continuous fluid delivery using a Masterflex L/S variable speed roller pump at 0.6 ml/min. Each DET cycle was composed of de-ionized water (resistivity 18.2 mΩ/cm) at 4 °C for 24 h, 4% sodium deoxycholate (Sigma) at room temperature (RT) for 4 h, and 2000kU DNase-I (Sigma) in 1m NaCl (Sigma) at RT for 3 h. Up to 4 cycles were carried out.

#### 2.2.4 Protocol D (IT/IN)

The trachea was perfused with intermittent fluid delivery using a syringe pump. Simulating the inspiratory cycle, each insufflation of the syringe pump was followed up by a withdrawal of the liquid that was infused. Four consecutive insufflations (each lasting 30 s) of each solution (de-ionized water (resistivity 18.2 mΩ/cm), 4% sodium deoxycholate (Sigma), and 2000kU DNase-I (Sigma) in 1m NaCl (Sigma) comprised one DET cycle. Up to 9 cycles were carried out.

In all the protocols after each treatment cycle the constructs were preserved at 4 °C, in PBS/AA and analyzed with the following methodologies.

### 2.3 Histology and immunostaining analysis

Samples were fixed for 24 h in 10% neutral buffered formalin solution in PBS (pH 7.4) at RT. Subsequently they were washed in distilled water (dH_2_O), dehydrated in graded alcohol, embedded in paraffin and sectioned at 5 μm. Tissue slides were stained with hematoxylin and eosin (H&E) (Leica, Germany), Masson's trichrome (MT), (Leica, Raymond A Lamb, BDH Chemicals Ltd), elastin Van Gieson (EVG) (VWR, Leica, Raymond A Lamb), and alcian blue (AB) (BDH Chemicals Ltd, Cellpath Ltd) stains. For immunofluorescence analysis, primary antibodies were used against laminin (Abcam, UK), collagen I (Abcam, UK), and collagen III (Abcam, UK) respectively, at dilutions of 1:100. The slides were visualized using confocal microscopy (Carl Zeiss).

### 2.4 DNA quantification

DNA was isolated using a tissue DNA isolation kit (PureLink Genomic DNA MiniKit, Invitrogen) following the manufacturer's instructions. Briefly, the samples were digested overnight using Proteinase K and a digestion buffer. DNA samples were purified using alcohol washes and measured spectrophotometrically (Nanodrop). Optical densities at 260 nm and 280 nm were used to estimate the purity and yield of nucleic acids.

### 2.5 GAG quantification

The sulfated GAG (GAG) content of native tissue and acellular matrices was quantified using the Blyscan GAG Assay Kit (Biocolor, UK). In brief, 50 mg of minced wet tissue was weighed and placed in a micro-centrifuge tube containing 1 ml of Papain digestion buffer and incubated in a water bath at 65 °C for 18 h, with occasional tube removal and vortexing. Aliquots of each sample were mixed with 1,9-dimethyl-methylene blue dye and reagents from the GAG assay kit. The absorbance at 656 nm was measured spectrophotometrically (Tecan Infinity) and compared to standards made from bovine tracheal chondroitin-4-sulfate to determine the GAG content.

### 2.6 Elastin quantification

The elastin content of native tissue and acellular matrices was quantified using the FASTIN elastin assay (Biocolor, UK) according to the manufacturer's instructions. Briefly, the samples were homogenized, and elastin was solubilized in 0.25 m oxalic acid. Two consecutive incubations were performed at 95 °C to ensure complete extraction of elastin. Extracts were incubated with 5,10,15,20-tetraphenyl-21H,23H-porphine tetrasulfonate (TPPS) dye, and absorbance was determined at 555 nm spectrophotometrically (Tecan Infinity). Elastin concentrations from a standard curve were used to calculate the elastin content of the tissue.

### 2.7 Scanning electron microscopy (SEM)

Samples were fixed in 2.5% glutaraldehyde in 0.1 m phosphate buffer and left for 24 h at 4 °C. Following washing with 0.1 m phosphate buffer, they were cut into segments of approximately 1 cm length and cryoprotected in 25% sucrose, 10% glycerol in 0.05 m PBS (pH 7.4) for 2 h, then fast frozen in Nitrogen slush and fractured at approximately −160 °C. The samples were then placed back into the cryoprotectant at room temperature and allowed to thaw. After washing in 0.1 m phosphate buffer (pH 7.4), the material was fixed in 1% OsO_4_/0.1 m phosphate buffer (pH 7.3) at 3 °C for 1½ hours and washed again in 0.1 m phosphate buffer (pH 7.4). After rinsing with dH_2_O, specimens were dehydrated in a graded ethanol-water series to 100% ethanol, critical point dried using CO_2_ and finally mounted on aluminum stubs using sticky carbon taps. The material was mounted to present the fractured surfaces across the parenchyma to the beam and coated with a thin layer of Au/Pd (approximately 2 nm thick) using a Gatan ion beam coater. Images were recorded with a Jeol 7401 FEG scanning electron microscope.

### 2.8 Transmission electron microscopy

Lung samples were cut into segments having a wall of approximately 1 cm in length. After washing in 0.1 m phosphate buffer (pH 7.4), they were fixed in 1% OsO_4_/0.1 m phosphate buffer (pH 7.3) at 3 °C for 1½ hours then washed in 0.1 m phosphate buffer (pH 7.4). Specimens were stained en bloc with 0.5% uranyl acetate in dH_2_O at 3 °C for 30 min, rinsed with dH_2_O, dehydrated in a graded ethanol-water series and infiltrated with Agar 100 resin and then hardened. Sections measuring 1 μm were cut and stained with 1% toluidine blue in dH_2_O for light microscopy. A representative area was selected and sections were cut at 70–80 nm using a diamond knife on a Reichert ultra-cut E microtome. Sections were collected on 200-mesh copper, coated slot grid and stained with uranyl acetate and lead citrate. Images were recorded with a Joel 1010 transition electron microscope.

### 2.9 Vascular network imaging

Vascular network imaging was carried out on rat scaffolds that were decellularized using protocol D. Vascular access was obtained by cannulating the pulmonary arteries via the right ventricle. For imaging of the vascular tree, 1% Trypan Blue (Sigma–Aldrich) was perfused into the scaffold at a rate of 2 ml/min. An iPhone 4S (Apple, US) was used to film the infusion and iMovie to separate stillshots.

### 2.10 Morphometric analysis

For morphometric analysis the standards for quantitative assessment of lung structure by the American Thoracic Society and European Respiratory Society were followed [17]. Fresh and decellularized lungs were fixed with 2.5% glutaraldehyde (pH 7.4) by rapid intratracheal flow at a head pressure 20–25 cm above the highest point of the lung. This ensured homogeneous lung fixation that would preserve fine parenchymal architecture. Following fixation, airway inflation pressure was maintained for 24 h by tying off the trachea. The samples were then washed in dH_2_O, dehydrated in graded alcohol, embedded in paraffin and sections were prepared at 5 μm. Tissue slides were stained with hematoxylin and eosin (H&E) (Leica, Germany) and low power field images were taken (5×) of adjacent sections that had 20% overlap. The images were stitched together on Fiji using a method that, based on the Fourier Shift Theorem, computes all possible translations between images and finds the best configuration of overlap [18]. Blending was implemented with maximum intensity. A random offset grid was placed on the images with an area of 5000 μm^2^. 20 fields were randomly selected and processed using point-counting to calculate fractional volumes of lung parenchyma (Vv(*p*)), alveolar space (Vv(a)), conducting airway space (Vv(c)), total airspace (Vv(A)) and alveolar septa (Vv(s)). Vv(*p*) was calculated in an indirect manner according to the suggestions of the American Thoracic Society task force on quantitative lung measurements [17]. Non-parenchymal volume (Vv(non-p)), that is, bronchioles, vessels, interlobular septa and lymph nodes was counted instead as it constituted a smaller fraction (about 10%):

Vv(p,L)=1−P(non−p)/P(L)

### 2.11 Alveolar size analysis

A total of 6 images per group (fresh vs. scaffold) containing 650 alveoli each were analyzed so as to examine the difference in alveolar dimension. To this aim, for each image the alveoli were segmented and their boundaries analytically obtained by means of an automated procedure as previously described [19,20]. Subsequently, the shape data were collected, the alveolar area computed and the two groups statistically compared.

### 2.12 Biomechanical testing

Pulmonary function was measured using the forced oscillation technique as previously described [21]. Excised rat lungs were cannulated with a 16 gage blunted needle and attached to a computer controlled piston ventilator (Flexivent, SCIREQ Inc. Montreal, QE, Canada). Lungs were ventilated at 90 breaths/minute, with a tidal volume of 2.0 ml, and a positive end expiratory pressure (PEEP) of 3 or 6 cm dH_2_O. Pressure was measured at the airway opening and volume is measured as the displacement of the piston of the ventilator. Lung mechanics were measured by applying a 2 s perturbation every 10 s for a total of 10 readings at each PEEP setting. These measurements involve interrupting mechanical ventilation while oscillatory flow signals are applied to the lung by the ventilator. The flow oscillations are non-damaging, having amplitudes less than or equal to normal tidal volume, and may contain small-amplitude frequency components up to 20 Hz. Multiple linear regression was used to fit measured pressure and volume in each individual mouse to the model of linear motion of the lung [22,23]. Model fits that resulted in a coefficient of determination less than 0.80 were excluded.

### 2.13 Chicken chorioallantoic membrane (CAM) angiogenic assay

To evaluate the angiogenic properties of the decellularized intestinal tissue in vivo we used the CAM assay as previously described [24,25]. Fertilized chicken eggs (Henry Stewart and Co., UK) were incubated at 37 °C and constant humidity. At 3 days of incubation an oval window of approximately 3 cm in diameter was cut into the shell with small dissecting scissors to reveal the embryo and CAM vessels. The window was sealed with tape and the eggs were returned to the incubator for a further 5 days. At day 8 of incubation, 1 mm diameter acellular lung matrices (both rat and sheep samples), or polyester used as a negative control, were placed on the CAM between branches of the blood vessels. Samples were examined daily until 7 days after placement wherein they were photographed in ovo with a stereomicroscope equipped with a Camera System (Leica) to quantify the blood vessels surrounding the matrices. Blood vessels less than 10 μm in diameter converging toward the tissues were counted by blinded assessors (*n* = 5), with the mean of the counts being considered.

## 3 Results

**Fig. 1:**
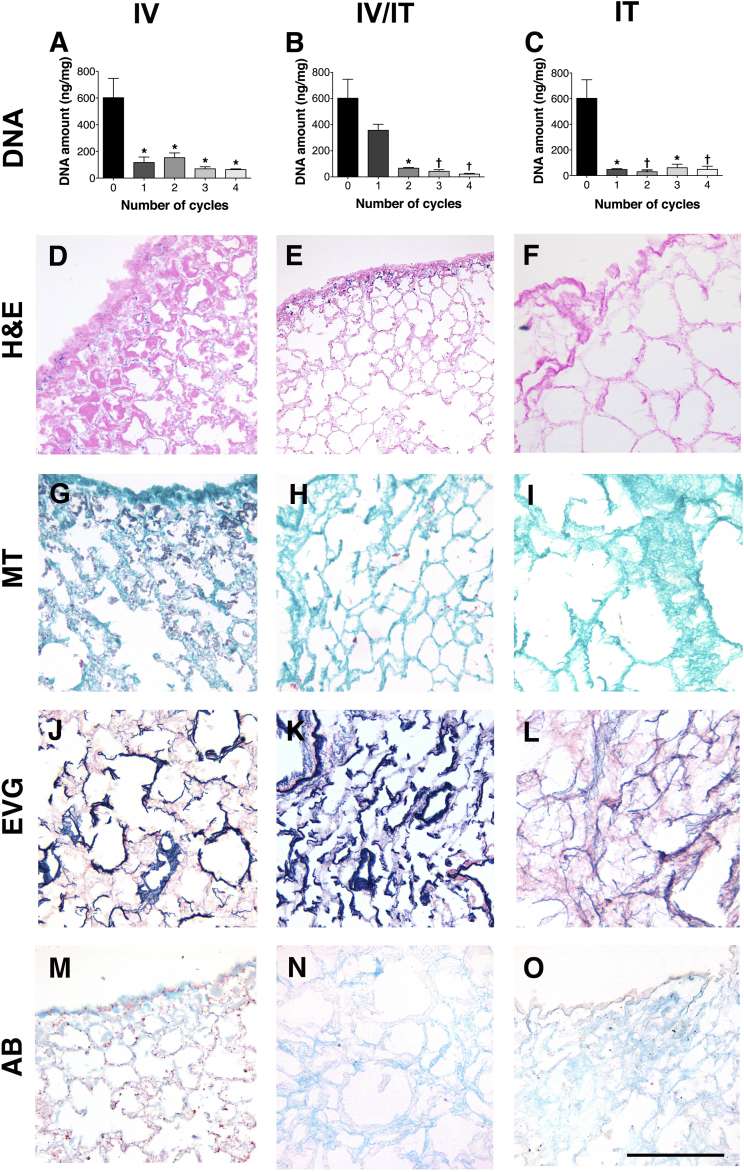
Decellularization via the vascular route reduces DNA (*p* < 0.05), following 1 cycle of treatment (A), with histological examination demonstrating an incomplete decellularization with nuclear and cytoplasmic material present (D, G, J, M). Further cycles do not reduce DNA significantly further. Decellularization from both vascular and tracheal routes leads to a reduction in DNA (*p* < 0.05) after 2 cycles with no significant decrease in 3 and 4 cycles (B). Histology confirms the removal of almost the entirety of nuclear and cytoplasmic material, with weak cellular staining being present on the lobar edges (E, H). Staining with with EVG and AB confirm a preservation of elastin and GAG (K, N). Decellularization via the tracheal route reduces DNA (*p* < 0.05) after only 1 cycle of treatment (C). Histology staining confirms an absence of nuclear material (F, I) and maintenance of elastin and GAG (L, O); IV: Intravascular, IV/IT: intravascular/intratracheal, IT: intratracheal, H&E: hematoxylin and eosin, MT: Masson's trichrome, EVG: elastin Van Gieson, AB: alcian blue, *: *P* < 0.05, †: *P* < 0.01, all statistical comparisons to fresh tissue, scale bar: 100 μm.

Decellularization using an intravascular approach from the pulmonary artery led to a reduction in DNA from 602 ± 145 ng/mg to 114 ± 22 ng/mg (*p* < 0.05), following 1 cycle of treatment (Fig. 1A). The incomplete decellularization that was also evident by the presence of nuclear and cytoplasmic material in H&E and MT staining (Fig. 1D, G), was not improved with further cycles of treatment. Decellularization using a combined intravascular-intratracheal method led to a reduction in DNA from 602 ± 145 ng/mg to 357 ± 46 ng/mg (ns) after 1 cycle and 67 ± 4 ng/mg (*p* < 0.05) after 2 cycles with no significant decrease in 3 and 4 cycles (Fig. 1B). Histology confirmed the removal of almost the entirety of nuclear and cytoplasmic material, with weak cellular staining being present on the lobar edges (Fig. 1E,H). Similar to the results with intravascular decellularization, staining with EVG and AB confirmed a preservation of elastin and GAG (Fig. 1J, K, M, N). Decellularization via the intratracheal route reduced DNA from 602 ± 145 ng/mg to 47 ± 9 ng/mg after only 1 cycle of treatment (*p* < 0.05) (Fig. 1C). H&E and MT staining confirmed an absence of nuclear material (Fig. 1F,I). GAG and elastin were maintained as shown by EVG and AB staining (Fig. 1L, O).

**Fig. 2:**
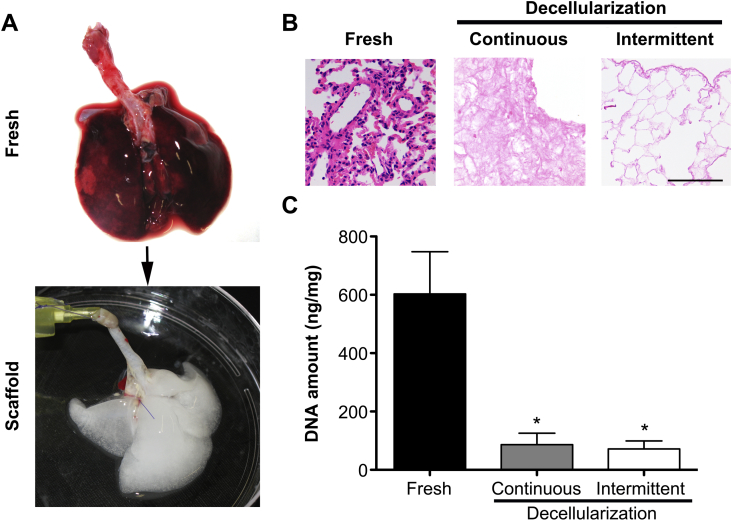
Decellularization using an intermittent intratracheal decellularization methodology yields an acellular scaffold that is macroscopically transparent (A) in a shorter time-interval when compared to the continous intratracheal protocol. Cellular material is removed equally well to the continuous methodology as evident by H&E staining and DNA quantification; H&E: hematoxylin and eosin, *: *P* < 0.05, all statistical comparisons to fresh tissue, scale bar: 100 μm.

Having established that intratracheal decellularization was superior to both vascular and combined approaches, we investigated whether intermittent inflation rather than continuous perfusion would further improve the decellularization technique. Indeed, decellularizing using an intermittent intratracheal inflation approach that mimics lung inspiration and expiration yielded an acellular scaffold that was macroscopically transparent following 9 cycles of treatment (Fig. 2A). One cycle of decellularization using this methodology lasts approximately 15 min, with 9 cycles lasting around 3 h. This compares very well to the continuous method of decellularization, in which one cycle comprises 31 h. Histological examination of the scaffolds obtained using intermittent intratracheal inflation demonstrated an improved preservation of the pulmonary architecture with inflated alveolar ducts and alveoli, and walls consisting of a solid collagenous network, in contrast to collapsed alveoli and thinned-out collagen in continuous intratracheal inflation (Fig. 2B). DNA was reduced from 602 ± 145 ng/mg to 71 ± 28 ng/mg in intermittent decellularization that was not significantly different to continuous decellularization (Fig. 2C).

**Fig. 3:**
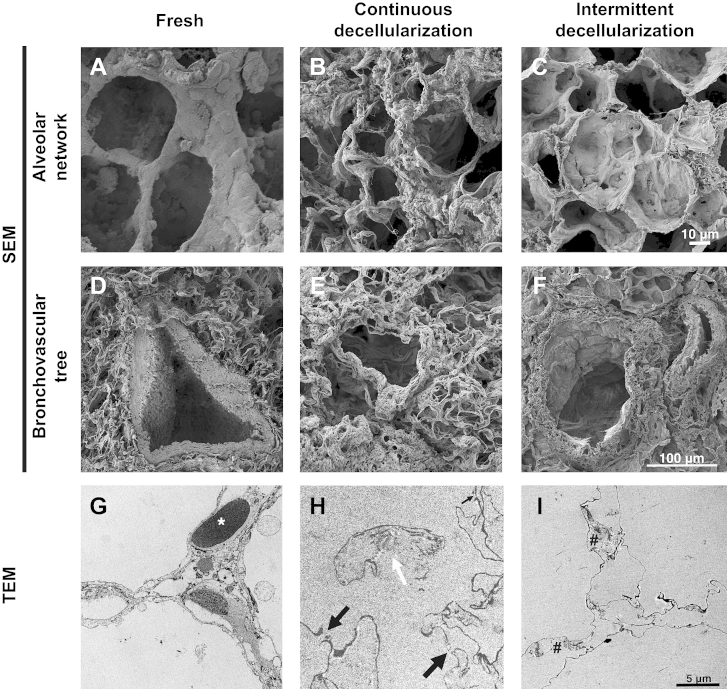
SEM demonstrates that in decellularized lung obtained with continuous inflation the bronchovascular tree (A, B) and alveolar network (D, E) are maintained but in a poor manner with weak collagen fibrils. Scaffolds obtained with intermittent intratracheal inflation show a complete preservation of bronchopulmonary structures (A, C), alveolar ducts and alveolar network (D, F) with an absence of cell nuclei. TEM of fresh lung shows the alveolar-capillary junction with red blood cells in close contact to the alveoli (asterisk, G), which following continuous decellularization, ruptures at a number of areas (black arrows, H), with exposure of the collagen fibers to the alveolus (white arrow, H). In sharp contrast, the intermittent approach leads to maintenance of the basement membrane and collagen fibers (I); SEM: scanning electron microscopy, TEM: transmission electron microscopy.

SEM of lungs that were decellularized intratracheally, both in a continuous and an intermittent manner, supported these results. Interestingly, decellularized lung obtained with continuous inflation showed a scaffold in which the bronchovascular tree and alveolar network were maintained but in a poor manner following 1 cycle, due to the loss of connective tissue from the pulmonary ECM (Fig. 3B,E). In comparison to this, intermittent inflation produced acellular scaffolds in which the bronchopulmonary structures, alveolar ducts and alveolar network were completely preserved with an absence of cell nuclei when examined at higher magnifications (Fig. 3C,F). TEM of fresh lung showed the alveolar-capillary junction with red blood cells in close contact with the alveoli (asterisk, Fig. 3G). Following continuous decellularization, the basement membrane had ruptured at a number of areas (black arrows, Fig. 3H) and the collagen fibers were exposed to the alveolus (white arrow, Fig. 3H). The intermittent approach led to maintenance of the basement membrane and collagen fibers (Fig. 3I).

**Fig. 4:**
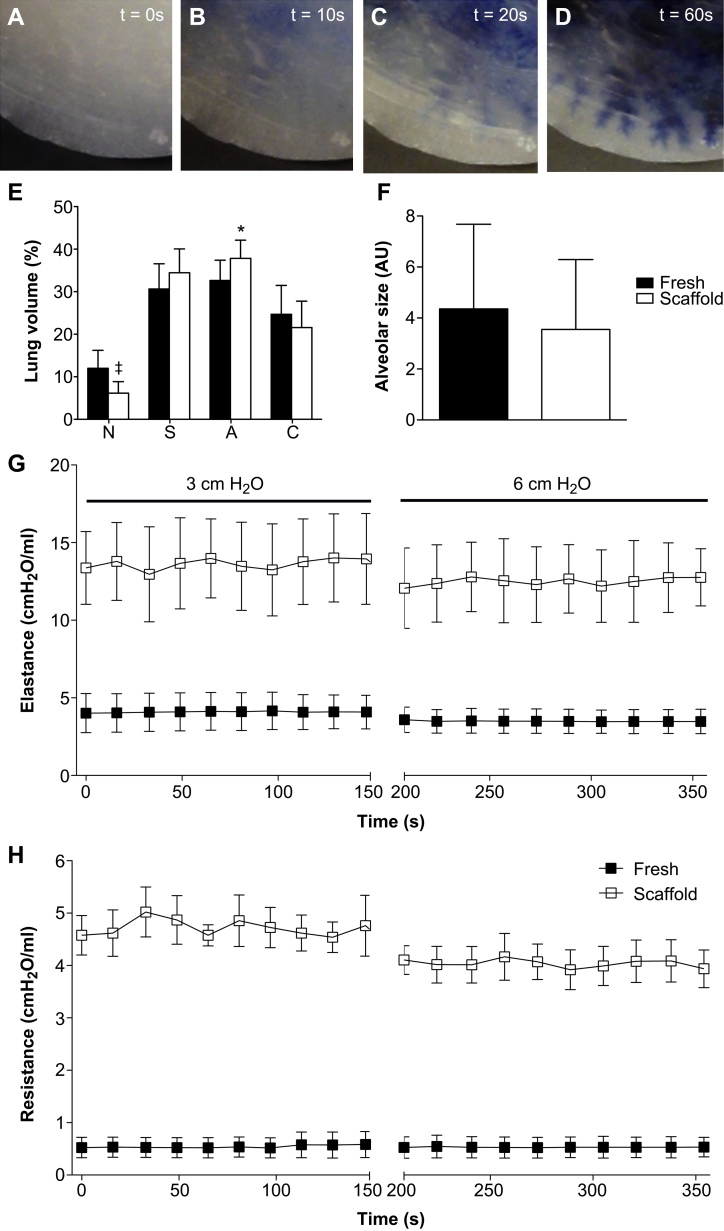
Pulsatile trypan blue injection of the pulmonary artery demonstrates progressive dye distribution from the central areas of the scaffold (A, B) to the periphery where fine branching of the capillaries is visible (C, D). Morphometry analysis of fresh lung and acellular scaffold (E) shows a significant decrease in non-parenchymal fractional volume (*P* < 0.001) and a corresponding increase in alveolar fractional volume (*P* < 0.05). Quantitative analysis of alveolar size indicates no differences in alveolar dimension following decellularization (F). Decellularized rat lungs show significantly increased airway elastance (G) and elastance (H) at positive end expiratory pressures (PEEP) of 3 and 6 cm H_2_O (*p* < 0.05); N: non-parenchymal fractional volume, S: septal fractional volume, A: alveolar space fractional volume, C: conducting airway fractional volume, AU: arbitrary units, *: *P* < 0.05, ‡: *P* < 0.001, all statistical comparisons to fresh tissue, scale bar: 100 μm.

We assessed the alveolar and vascular networks of the scaffold obtained with intermittent decellularization as markers of micro-architecture maintenance. We injected the pulmonary artery with trypan blue whilst performing time-lapse microscopy (Suppl. mov. 1). Following injection, the dye distributed in the central areas of the scaffold first (Fig. 4A). As dye approached the periphery the fine branching of the vascular tree could also be seen (Fig. 4C,D). The clear edge of the lung was a sign of the lack of leakage from the scaffold (Fig. 4D). Morphometry analysis of fresh lung and acellular scaffold (Fig. 4E) demonstrated maintenance of the pulmonary components alongside a decrease in non-parenchymal volume from 12.1% to 6.15% (*P* < 0.0001). A significant increase was also noted in alveolar fractional volume with a rise from 32.6% to 37.8% (*P* < 0.05). A quantitative analysis of alveolar size showed that the decellularized alveolar area is reduced together with the variability, therefore the decellularized area values are included in those of the fresh samples (Fig. 4F). This suggests that the decellularization process does not introduce new features in terms of alveolar dimension.

Supplementary data related to this article can be found online at http://dx.doi.org/10.1016/j.biomaterials.2013.05.015.

The following is the Supplementary data related to this article:

Suppl. Mov. 1 Time-lapse imaging of the lung scaffold following trypan blue injections.

Both airway elastance (Fig. 4G) and resistance (Fig. 4H) were determined at positive end expiratory pressures (PEEP) of 3 and 6 cm H_2_O. Decellularized rat lungs showed significantly increased airway resistance and elastance at both PEEP levels (*p* < 0.05). The resistance values were 0.5 ± 0.3 cm H_2_O and 4.4 ± 0.7 cm H_2_O for native lung and decellularized lung, respectively. The elastance values were 3.8 ± 1.6 cm H_2_O and 13.1 ± 1.7 cm H_2_O for native lung and decellularized lung, respectively. No significant difference was observed based on the change in PEEP, however there was a trend towards lower elastance at a PEEP of 6 cm H_2_O for decellularized lungs.

**Fig. 5:**
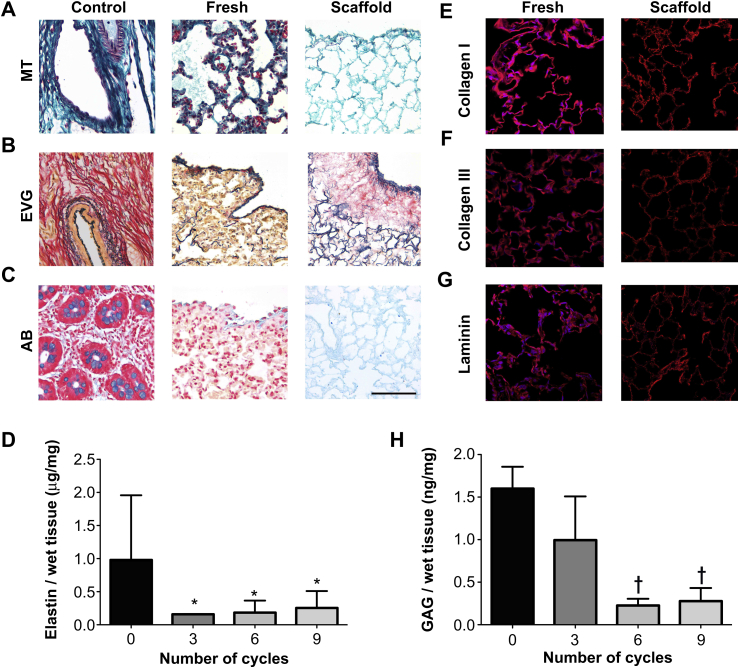
MT staining demonstrates a hierarchical alveolar network, with preservation of collagen in both central and peripheral areas (A). EVG and AB staining show preservation of elastic fibers and GAG (B, C). Immunofluorescence for collagen I, collagen III and laminin shows maintenance of the ECM components with no DAPI staining in the scaffolds when compared to fresh tissue (E–G). GAG quantification demonstrates a reduction to 19% of the fresh value (*P* < 0.01) (H); MT: Masson's trichrome, EVG: elastin Van Gieson, AB: alcian blue, GAG: glycosaminoglycans *: *P* < 0.05, †: *P* < 0.01, all statistical comparisons to fresh tissue, scale bar: 100 μm.

MT staining of the scaffolds obtained using intermittent inflation demonstrated a well-structured alveolar network, with preservation of collagen in both central and peripheral areas (Fig. 5A). EVG staining indicated preservation of peripheral, axial and septal elastic fibers (Fig. 5B). Elastin quantification demonstrated a major variability in the elastin content of fresh samples with levels of 0.98 ± 0.98 ng/mg and a progressive non-significant reduction to 0.3 ± 0.3 ng/mg at 9 cycles of treatment (Fig. 5D). AB staining confirmed the preservation of GAG in the lung scaffold following decellularization (Fig. 5C). The preservation of GAG is confirmed with quantification with content decreasing from 1.6 ± 0.3 ng/mg to 0.3 ± 0.2 ng/mg, a reduction to 19% of the fresh value (*P* < 0.01) (Fig. 5H). Immunofluorescence for collagen I, collagen III and laminin showed good maintenance of the components of the ECM together with a complete absence of nuclear material as evidenced by DAPI staining (Fig. 5E–G).

**Fig. 6:**
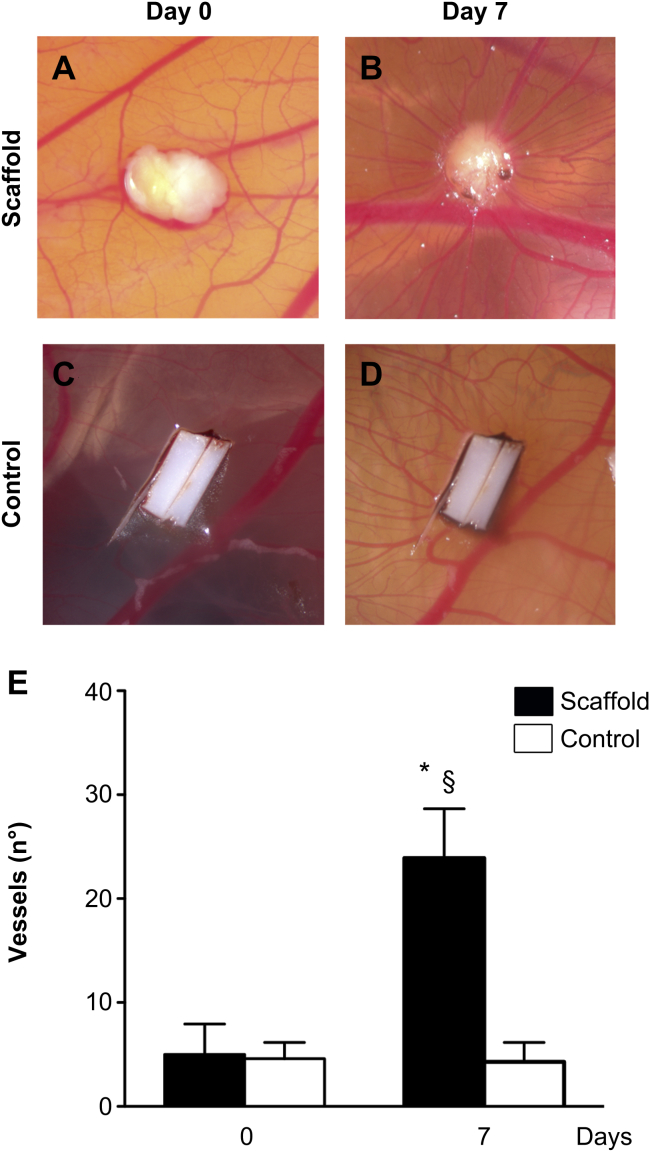
The angiogenic properties of the scaffold are demonstrated in vivo following placement on top of the chicken chorioallantoic membrane. On days 7 after implantation, the number of vessels converging towards the lung matrices is significantly increased in comparison to the same samples at day 0 (*P* < 0.05) and to the polyester membrane that was used as a negative control (*P* < 0.01) (A). Representative images of lung tissue placed in ovo at 0 (B), and 7 days (C) Of incubation, indicate attraction of blood vessels that, in a cogwheel fashion, seem to penetrate the tissue. The polyester control, at the same time-points, has no effect on vascular development around it (D,E); *: *P* < 0.05, comparison to scaffold, day 0 §: *P* < 0.01, comparison to control, day 7.

To evaluate the in vivo potential of the scaffold, segments were placed on top of the chicken chorioallantoic membrane. The scaffolds integrated well with the developing environment of the chicken egg. Representative images of lung tissue placed in ovo at 0 (Fig. 6A), and 7 days (Fig. 6B) of incubation, demonstrated attraction of blood vessels that, in a spoke-wheel pattern, seem to penetrate the tissue. The polyester control, at the same time-points, had no effect on vascular development around it (Fig. 6C,D). Seven days after implantation, the number of vessels converging towards the lung matrices was significantly increased in comparison to the same samples at day 0 (*P* < 0.05) and to the polyester membrane that was used as a negative control (*P* < 0.01) (Fig. 6E).

**Fig. 7:**
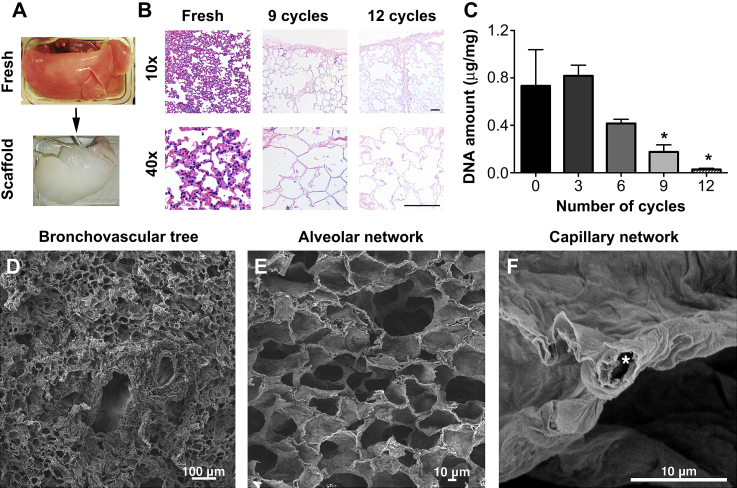
The decellularized sheep scaffold represents a solid mass that maintains the architecture of the original lung, whilst being transparent indicating a lack of cells (A). Decellularization is achieved at 12 cycles with H&E (B) and DNA analysis (*P* < 0.05) confirming the absence of nuclear material. SEM shows no visible cells and preservation of bronchovascular structures (D), alveolar ducts and network (E), as well as the capillaries that are in close contact with the alveoli (asterisk, F); *: *P* < 0.05, all statistical comparisons to fresh tissue, scale bar: 100 μm.

**Fig. 8:**
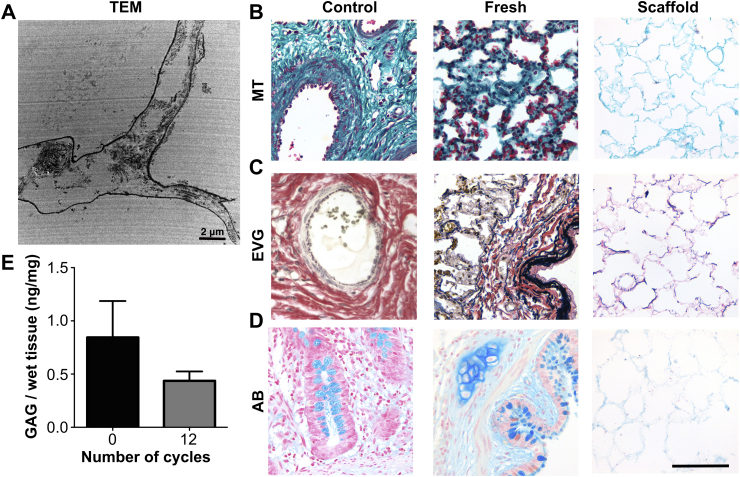
TEM shows maintenance of the basement membrane and collagen fibrils (A). MT staining indicates, similarly to the rat scaffold, a hierarchical collagenous network (B). EVG and AB staining display preservation of elastin and GAG (C, D). GAG quantification demonstrates a non-significant decrease in GAG values when compared to fresh tissue (E); H&E: hematoxylin and eosin, MT: Masson's trichrome, EVG: elastin Van Gieson, AB: alcian blue, GAG: glycosaminoglycans, SEM: scanning electron microscopy, TEM: transmission electron microscopy, *: *P* < 0.05, †: *P* < 0.01, all statistical comparisons to fresh tissue, scale bar: 100 μm.

To assess whether the described methodology could be used established for human lungs, we examined its' efficiency in decellularizing sheep lungs. The macroscopic image of the decellularized scaffold represents a solid mass that maintains the architecture of the original lung, whilst being transparent indicates a lack of cells (Fig. 7A). Decellularization was achieved at 12 cycles, indicated by H&E (Fig. 7B) and DAPI staining (data not shown), which confirmed the absence of nuclear material. DNA was reduced from 733 ± 305.4 ng/mg to 27.6 ± 9.4 ng/mg (*p* < 0.05) following 12 cycles (Fig. 7C). SEM showed, similar to the rat scaffold, that there were no visible cells and there was an excellent preservation of bronchovascular structures (Fig. 7D), alveolar ducts and network (Fig. 7E), as well as the capillaries that are in close contact with the alveoli (asterisk, Fig. 7F). TEM indicated that following decellularization there was complete removal of all cellular material and maintenance of the basement membrane (Fig. 8A). MT staining indicated, similarly to the rat scaffold, a hierarchical collagenous network, in both central as well as peripheral areas (Fig. 8B). EVG staining displayed an excellent preservation of elastin (Fig. 8C). On the other hand, AB staining showed weaker staining compared to the rat scaffold (Fig. 8D). However, GAG quantification demonstrated preservation of GAG, with a non-significant decrease from 0.84 ± 0.3 ng/mg to 0.44 ± 0.1 ng/mg, a reduction to 52% of the fresh value (Fig. 8E).

## 4 Discussion

Lung tissue engineering has had limited successful applications because of the difficulties associated with recreating the intricacy of the bronchioalveolar network and the alveolus-capillary interface. Given the range of scaffolds currently available, the category that holds the most potential in recreating this complex three-dimensional architecture is that of natural acellular matrices. However, efforts to produce an optimal acellular matrix have used long and harsh decellularization protocols [26,27]. In this study, using a simple, clinically applicable methodology, we were able to achieve complete decellularization of both rat and sheep lungs whilst maintaining their microarchitecture.

We first established that intra-tracheal was the optimal entry site for decellularization which was obtained in just 1 cycle. On the contrary intravascular access did not lead to complete decellularization at 4 cycles of treatment, and the combination of vascular and tracheal access required 2 cycles. Early protocols used simple immersion of the lungs in a series of detergents for an extended period of time until cells were removed [28]. More recently, the focus has shifted towards dynamic protocols, in which the trachea and the pulmonary artery are used to infuse the decellularization solutions [13,14,29,30]. Other laboratories have used both ports of entry with complex systems, which, if translated to clinical practice, could be prone to contamination. Similar to our findings, Cortiella et al. [31] omitted the vascular access and used only the trachea to decellularize rat lungs. However, while they took 7 weeks to obtain efficient decellularization, using our detergent-enzymatic method (deionized water, sodium deoxycholate, DNase) we were able to reduce this to just 31 (continuous perfusion) and 3 (intermittent perfusion) hours respectively. Even in a large animal model such as the sheep we were able to demonstrate that complete decellularization was achieved within 6 h using an intermittent perfusion.

Importantly, all approaches retained ECM components, even though, using continuous perfusion, pulmonary micro-architecture maintenance was not optimal as evidenced by areas of tearing and collapse. We improved the maintenance of the scaffold architecture using intermittent inflation with DET, mimicking an inspiratory/expiratory system. Interestingly, while histology did not demonstrate any differences, SEM revealed a structure that had weak alveolar walls with thinned-out collagen. With all of the articles on lung decellularized scaffolds not demonstrating results from SEM at a high magnification to assess structure, it may be important for future studies to utilize this methodology as a qualitative assessment for refining the decellularization protocol.

Morphometric analysis of fresh lung and acellular scaffold indicated a decrease in non-parenchymal volume and an increase in alveolar fractional volume. The former may be explained due to the inability to identify acellular non-parenchymal structures such as blood vessels and lymph nodes during point-counting of the scaffold. The alveolar fractional volume increase, which was previously encountered in SDS-treated scaffolds [13], may be attributed to the higher possibility of point-counting the alveolar space when the volume occupied by alveolar cells (included in the alveolar septal fractional volume) is lost due to decellularization. We were interested to assess whether the increase in alveolar fractional volume could be due to an alteration in its' shape brought about by decellularization. A non-stereological quantitative evaluation of the alveolar size in fresh and decellularized lungs highlighted that in the two groups the alveolar dimension is comparable. Similarly to previous reports, the decellularized rat lungs showed similar increases in airway resistance and elastance [29,31,32]. However, no negative values for resistance were encountered, as has been the case with CHAPS- and SDS-treated lungs, an occurrence that was attributed to leakage during the oscillatory perturbations. Interestingly, the increase in elastance was PEEP-independent, with no significant differences observed between 3 and 6 cm H2O. Such results may demonstrate that the alveoli have collapsed and no further tension may recruit them. Addition of surfactant in mouse decellularized lungs reduced elastance and brought about PEEP-responsiveness and may be considered in the future for decellularized human lungs [29]. Nevertheless, relevant information obtained through mechanical analysis may be important for future studies to assess the biomechanics of the decellularized lungs and monitor whether preservation of the ECM components has any effect on scaffold elastance and resistance.

These properties may eventually be regulated by the preservation of the ECM components. Pulmonary ECM is mainly composed of collagen, elastin, laminin, GAG, fibronectin, entactin and tenascin Ref. [33]. We corroborated the results in preservation of architecture and presence of elastin and GAG. The levels of elastin preservation are similar to published protocols and are highly important in the eventual pulmonary dynamics of the repopulated scaffold. GAG maintenance is important, as it has been suggested that this could favorably influence cell attachment and proliferation. Immunofluorescence for laminin, collagen I and III in the scaffolds demonstrated preservation compared to fresh tissue, whilst there was an absence of cellular material as evidenced by DAPI. Other components, such as vascular pro-angiogenic factors were evaluated using an established in vivo CAM assay. When placed on top of the chicken chorioallantoic membrane, vessels were seen converging towards the scaffold in a circular fashion. This could be extremely relevant for endothelial cell seeding and neo-capillary formation in the engineered lung. All these characteristics are important but irrelevant if not reproducible in a lung that has similar dimensions and anatomical characteristics to the human one.

Previous work done for other organs, such as the kidney [34], liver [35], heart [36], trachea [7,37,38] and esophagus [39] has scaled up scaffolds for clinical translation. We thus observed that this methodology could be scaled up to a large animal model for the lung. Decellularization was achieved in a higher number of cycles as would be expected, but, similarly to the experiments in rat lung, the decellularization process removed almost the entirety of nuclear and cytoplasmic material whilst maintaining the pulmonary architecture. SEM confirmed the preservation of capillaries that were fractionated on the interface with the alveoli. Relevant to the clinical application of this approach was the fact that with these larger lungs there was an improved preservation of the ECM components. For example, TEM indicated an improved conservation of large collagen bundles, whilst elastin and GAG quantification demonstrated non-significant reductions to the levels in the scaffold when compared to fresh tissue.

The golden chalice of this work is a scaffold populated by recipient cells, able to be transplanted and to function. Future experiments will be conducted in bioreactors to explore re-population of the scaffold with lung progenitor cells. Subsequently, we envisage transplantation of the scaffolds following pneumonectomy in animal models.

## 5 Conclusions

We have previously published on the benefits of using the detergent-enzymatic treatment (DET) for the creation of tracheal, intestinal and esophageal natural acellular matrices. We have now established a methodology that produces a pulmonary natural acellular matrix that preserves the microarchitecture of the tissue both in the vascular and bronchial counterparts. The production of the scaffold is a quick process at less than 3 h, and can be scaled up to a larger animal with similar results. Similarly to what we have shown for other organs, we were able to establish for the lung a quick protocol that avoids harsh chemicals that were deemed a necessity for complex organs thus far.
