# Supplementary figures and images for: Preservation of micro-architecture and angiogenic potential in a pulmonary acellular matrix obtained using intermittent intra-tracheal flow of detergent enzymatic treatment
Source: Biomaterials. 2013 Sep;34(28):6638–48. doi: 10.1016/j.biomaterials.2013.05.015 (PMC3988964; doi:10.1016/j.biomaterials.2013.05.015)

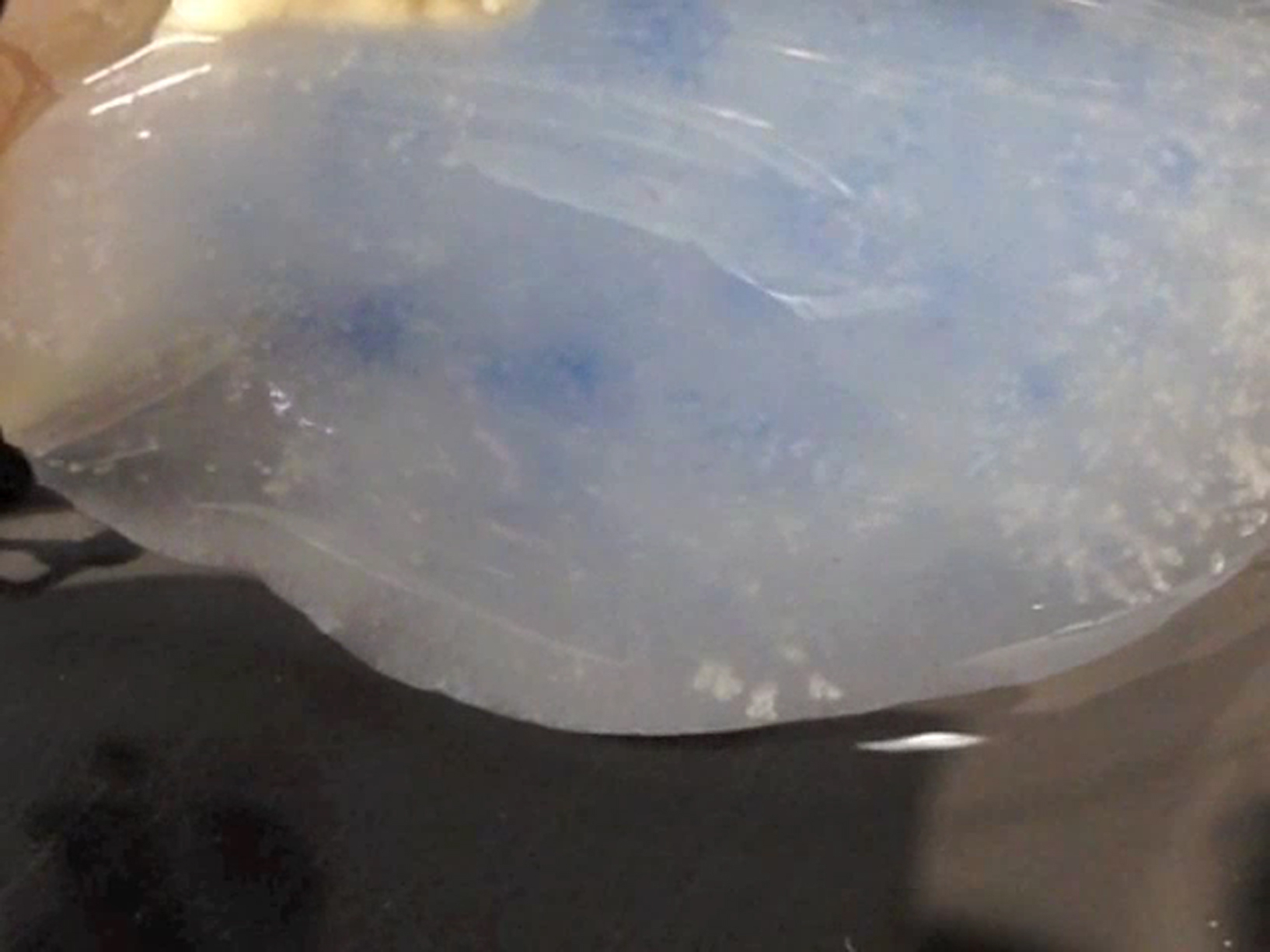

Supplement: Suppl. Mov. 1 — Time-lapse imaging of the lung scaffold following trypan blue injections. [file mmc1.jpg]
